# Effects of non-Newtonian viscosity on arterial and venous flow and transport

**DOI:** 10.1038/s41598-022-19867-1

**Published:** 2022-11-29

**Authors:** Sabrina Lynch, Nitesh Nama, C. Alberto Figueroa

**Affiliations:** 1 Department of Biomedical Engineering, University of Michigan, Ann Arbor, MI USA; 2 Department of Mechanical & Materials Engineering, University of Nebraska, Lincoln, NE USA; 3 Department of Surgery, University of Michigan, Ann Arbor, MI USA

**Keywords:** Biomedical engineering, Aortic diseases

## Abstract

It is well known that blood exhibits non-Newtonian viscosity, but it is generally modeled as a Newtonian fluid. However, in situations of low shear rate, the validity of the Newtonian assumption is questionable. In this study, we investigated differences between Newtonian and non-Newtonian hemodynamic metrics such as velocity, vorticity, and wall shear stress. In addition, we investigated cardiovascular transport using two different approaches, Eulerian mass transport and Lagrangian particle tracking. Non-Newtonian solutions revealed important differences in both hemodynamic and transport metrics relative to the Newtonian model. Most notably for the hemodynamic metrics, in-plane velocity and vorticity were consistently larger in the Newtonian approximation for both arterial and venous flows. Conversely, wall shear stresses were larger for the non-Newtonian case for both the arterial and venous models. Our results also indicate that for the Lagrangian metrics, the history of accumulated shear was consistently larger for both arterial and venous flows in the Newtonian approximation. Lastly, our results also suggest that the Newtonian model produces larger near wall and luminal mass transport values compared to the non-Newtonian model, likely due to the increased vorticity and recirculation. These findings demonstrate the importance of accounting for non-Newtonian behavior in cardiovascular flows exhibiting significant regions of low shear rate and recirculation.

## Introduction

Despite significant progress in clinical research and care, cardiovascular diseases remain the leading cause of death and disability worldwide^1^. Advances in both experimental and computational modeling techniques have led to an improved understanding of basic mechanisms underlying various cardiovascular diseases. In particular, numerous studies have implicated altered local hemodynamic metrics (e.g., vorticity, wall shear stress (WSS), etc.) in the initiation and progression of thrombosis and atherosclerosis^2–4^. Moreover, anatomical features such as aneurysms trigger complex local flow patterns, which lead to increases in residence time of biochemical species^5,6^. Therefore, computational models for cardiovascular disease research must account for the altered hemodynamics in image-based geometric models, specifically in areas of recirculation likely to experience complex transport phenomena.

An important aspect of a computational model is the constitutive assumption. Blood has been shown to exhibit shear-thinning behavior and has been described via various shear-dependent constitutive models such as the Carreau-Yasuda and Power-Law models^7^. Nevertheless, most computational hemodynamics studies have treated blood as a Newtonian fluid with uniform viscosity^8–10^. The use of a Newtonian assumption for blood is typically justified for large arteries with high shear rate flows. However, this assumption becomes questionable in regions exhibiting low shear rates and local flow recirculation such as aneurysmal vessels and veins. Therefore, it is crucial to incorporate shear rate dependent blood rheology for accurately describing the local hemodynamics in such cases.

Previous studies of non-Newtonian viscosity have often employed idealized vascular geometries^11^, despite the fact that the vessel anatomy significantly impacts blood flow dynamics by triggering complex local flow patterns. Several other studies have investigated the effects of non-Newtonian viscosity in healthy and diseased arterial models^3,12^. While some studies concluded that considering the shear thinning behavior of blood is significant^3,12–16^, others reported relatively minor differences in hemodynamics^17,18^. With regards to venous flows, most prior studies have considered a Newtonian assumption for blood^19,20^, and only a few contributions have considered non-Newtonian behavior^21^, albeit under idealized (constant) flow conditions. In addition, few previous studies of non-Newtonian viscosity have investigated the influence of non-Newtonian viscosity on either Eulerian or Lagrangian transport phenomena^22,23^, which plays a significant role in disease initiation.

Various constitutive models have been developed and calibrated against experimental data to describe the shear-thinning behavior of blood. Commonly used constitutive models include the Power-Law, Casson, Herschel-Bulkley, and Carreau-Yasuda models. The Power-Law fluid model employs two constants to describe a linear relationship between viscosity and shear-rate on a log-log scale. This model has an analytical solution which makes it useful for development purposes, but the Power-Law model fails to accurately model viscosity at very low and high shear rates, limiting its clinical applicability. The Casson and Herschel-Bulkley models accurately describe blood viscosity at intermediate to high shear-rates, but fail to accurately capture blood viscosity at lower shear-rates. The Carreau-Yasuda model approaches asymptotic values of the effective viscosity at zero and infinite shear, making it more applicable for clinical studies^12^.

In this work, we aimed to increase our understanding of the impact of blood rheology in complex arterial and venous flows, with significant regions of low shear rates. Towards that end, we implemented two different models of non-Newtonian viscosity within the cardiovascular hemodynamics modeling environment CRIMSON^24^: *(i)* a Power-Law model and *(ii)* the Carreau-Yasuda model. In addition to exploring the impact of non-Newtonian viscosity on traditional hemodynamic metrics such as velocity and wall shear stress, we aimed to examine other metrics such as near-wall mass transport and Lagrangian indices of accumulated shear.

The structure of this article is as follows. In “Methods” section, we provide an overview of the governing equations, constitutive models, and the two methods used to study the impact of non-Newtonian viscosity on transport. In “Results” section, we verify our implementation of the Power-Law model against an analytical solution in an idealized cylindrical geometry. Next, we investigate the effects of non-Newtonian rheology (using the Carreau-Yasuda model) in two representative three-dimensional, transient, image-based scenarios: (a) a thoracic aortic aneurysm model, and (b) a venous model of the inferior vena cava and iliac bifurcation. In each case, we report the differences between in-plane velocity, vorticity, WSS, mass transport, and Lagrangian indices of shear between the Newtonian and Carreau-Yasuda models.

## Methods

### Fluid dynamics

#### Governing equations

The strong form of the governing equations for an incompressible fluid in a three-dimensional bounded domain $\Omega \subset {\mathbf {R}}^3$ is given as

1 $$\begin{aligned} \rho \left( \frac{\partial {{\textbf {u}}}}{\partial t} + {{\textbf {u}}} \cdot \mathbf {\nabla } {{\textbf {u}}} \right)&= - \mathbf {\nabla } p + \mathbf {\nabla } \cdot \varvec{\tau }({{\textbf {u}}} )+{\mathbf {f}} , \end{aligned}$$

2 $$\begin{aligned} \mathbf {\nabla } \cdot {{\textbf {u}}}&= 0, \end{aligned}$$

where $\rho$ is the fluid density, *t* is the time, ${{\textbf {u}}}$ is the fluid velocity, *p* is pressure, ${\mathbf {f}}$ is the external body force per unit volume (set to zero), and $\varvec{\tau }$ is the viscous stress tensor. For a Newtonian, incompressible fluid, $\varvec{\tau }$ is defined as:

3 $$\begin{aligned} \varvec{\tau }&= 2 \mu {\mathbf {D}}, \end{aligned}$$

where $\mu$ is the Newtonian viscosity and ${\mathbf {D}}$ is the rate of deformation tensor defined as:

4 $$\begin{aligned} {\mathbf {D}}&:= \frac{\left( \mathbf {\nabla } {{\textbf {u}}} + \mathbf {\nabla } {{\textbf {u}}}^T\right) }{2}. \end{aligned}$$

For a non-Newtonian fluid $\varvec{\tau }$ can be written as:

5 $$\begin{aligned} \varvec{\tau }&= 2 {\mu }_\text {eff} ({\dot{\gamma }}) {\mathbf {D}}, \end{aligned}$$

where ${\dot{\gamma }}$ refers to the shear rate defined as

6 $$\begin{aligned} {\dot{\gamma }}= \sqrt{2{\mathbf {D}}:{\mathbf {D}}}, \end{aligned}$$

and ${\mu }_\text {eff}({\dot{\gamma }})$ is the constitutive shear rate function which describes the effective viscosity in terms of ${\dot{\gamma }}$.

#### Constitutive models of non-Newtonian viscosity

Various constitutive models have been developed and calibrated against experimental data to describe the shear-thinning behavior of blood. Table 1 presents the three constitutive models considered in this study and their associated parameter values, chosen from previous reports^3,7,25,26^: (a) Newtonian model, (b) Power-Law model, and (c) Carreau-Yasuda model. The Newtonian fluid model employs a constant, shear-independent viscosity ($\mu _{\mathrm {N}}$). In contrast, the Power-Law fluid model employs two constants, a flow consistency index $\delta$, and a flow behavior index *n*, to model the shear dependent blood viscosity. Lastly, the Carreau-Yasuda model employs five constants: a relaxation time $\lambda$, power indices *n* and *a*, and asymptotic values of the effective viscosity $\mu ({\dot{\gamma }})$, $\mu _0$ and $\mu _{\infty }$, at zero and infinite shear-rates, respectively.

**Table 1:** Constitutive models and the associated parameter values.

Constitutive models	Form	Parameter values
Newtonian	$\mu ({\dot{\gamma }}) = \mu _{\mathrm {N}}$	$\mu _{\mathrm {N}} = 0.0035$
Power-law	$\mu ({\dot{\gamma }}) = \delta {\dot{\gamma }}^{n-1}$	$\delta = 0.0147, \ n = 0.7755$
Carreau-Yasuda	$\mu ({\dot{\gamma }}) = \mu _{\infty } + \left( \mu _0 - \mu _{\infty } \right) \left( 1 + \left( \lambda {\dot{\gamma }}\right) ^a \right) ^{\frac{n-1}{a}}$	$\mu _{\infty } = 0.0035, \ \mu _0 = 0.16,$ $\lambda = 8.2, \ n = 0.2128,$ $a = 0.64$

The behavior of these three models for different shear rates is plotted in Fig. S1 of the Electronic Supplementary Information. The Power-Law model displays a linear response with a constant slope given by the flow behavior index *n* on a log-log scale. Values of $n > 1$ imply shear thickening behavior while values of $n < 1$ imply shear-thinning behavior. Conversely, the Carreau-Yasuda model is characterized by constant values of viscosity in both the low ($\mu _0$) and high ($\mu _{\infty }$) shear-rate limits. Between these limits, the viscosity varies in a nonlinear manner on the log-log scale.

### Transport analysis

A significant goal of this work is to understand the impact of non-Newtonian rheology on mass transport. Towards that goal, we considered two separate approaches to assess mass transport within a given flow field: *(i)* Lagrangian particle tracking, and *(ii)* Mass transport via advection-diffusion (AD).

#### Lagrangian particle tracking

The Navier-Stokes equations are typically solved in fixed Eulerian formulations. Consequently, the solution fields (velocity and pressure) do not directly provide insights on the path of particles traversing through the flow field. In contrast, a Lagrangian representation of a flow field can be used to describe the path, history of hemodynamic stresses, and residence time experienced by a particle in certain parts of the vasculature. In blood flow, constituents such as platelets are small enough in size to be reasonably approximated by massless particles advected by the flow. The path and the history of stress of these particles are important quantities to study processes such as platelet mechanical activation, a key process in thrombus formation^2,27^. Therefore, in this work, we considered a Lagrangian particle tracking method derived from the Eulerian solution fields to assess the cumulative shear experienced by massless particles^2,28^. Specifically, we introduced a number of particles with prescribed initial positions and integrated their Lagrangian trajectories from the Eulerian flow field solution using a fourth-order Runge-Kutta scheme. This Lagrangian tracking allowed us to assess the ‘platelet activation potential’ (PLAP), a metric that has been linked to thrombus formation in thoracic and abdominal aortic flows^2,29^. PLAP is a non-dimensional scalar index that represents the magnitude of shear rates that a particle accumulates while traveling through the fluid domain and is defined as

7 $$\begin{aligned} PLAP({{\textbf {x}}},t) = \int _{t-T}^t |{{\textbf {D}}}({{\textbf {x}}}({\tilde{t}}),{\tilde{t}})|d{\tilde{t}}, \end{aligned}$$

where $|{{\textbf {D}}}({{\textbf {x}}}({\tilde{t}}),{\tilde{t}})|$ is the Frobenius norm of the symmetric part of the spatial gradient of the velocity tensor, *t* is the current time, and *T* indicates how long the particle has been tracked.

#### Scalar mass transport via advection-diffusion (AD)

Eulerian formulations have also been widely used to study the mass transport of proteins or other chemical species in cardiovascular flows, in particular for thrombosis research^25,30,31^. In this approach, scalar AD equations are solved to obtain the spatio-temporal distribution of the concentration of the species. The strong form of the AD equations for mass transport in a three-dimensional bounded domain $\Omega \subset {\mathbb {R}}^3$ is given as

8 $$\begin{aligned} \frac{\partial c_i}{\partial t} + {{\textbf {u}}} \cdot \nabla c_i - \nabla \cdot \left( D_i \nabla c_i \right)&= r_i \qquad \text {in} \quad \Omega \quad \ \mathrm {for} \ i = 1, ..., \mathrm {number \ of \ scalars}, \end{aligned}$$

where $i = 1,\ ..., \mathrm {number \ of \ scalars}$ and $c_i$, $D_i$, and $r_i$ refer to the concentration, diffusion coefficient, reaction term(s) for the scalar *i*.

Given our interest in understanding the impact of blood rheology on velocity, in this work, we focused our attention on the advective component of scalar AD transport equations (Eq. 8), assuming a constant diffusivity throughout the entire flow field. Towards that goal, we prescribed concentrations of species through the inlet face(s) of the computational model under two different constitutive assumptions (Newtonian and Carreau-Yasuda), and solved the spatio-temporal concentration fields, to study potential discrepancies in mass transport in the lumen and near the vessel wall. Details of the scalar AD formulation used in this article can be found elsewhere^32^.

### Patient-specific models and boundary conditions

Two patient-specific geometries were considered: *(i)* a thoracic aortic aneurysm model, and *(ii)* a venous model of the inferior vena cava and iliac veins. Approval was obtained from the institutional review board (HUM00155491). To ensure a consistent comparison between the Newtonian and Carreau-Yasuda models, the parameter values listed in Table 1 were adopted. Specifically, following Marrero *et al.*^17^, the parameter $\mu _{\infty }$ was chosen to match the Newtonian viscosity ($\mu _{\infty }=\mu _{\mathrm {N}} = 0.0035~\text {Pa} \; \text {s}$). Therefore, both the Newtonian and Carreau-Yasuda models yield the same effective viscosity in the high shear rate limit. At low shear rates, the Newtonian model exhibits lower viscosity. Blood density was $1060~\text {kg}/\text {m}^3$ for both models.

#### Arterial model

**Figure 1:**
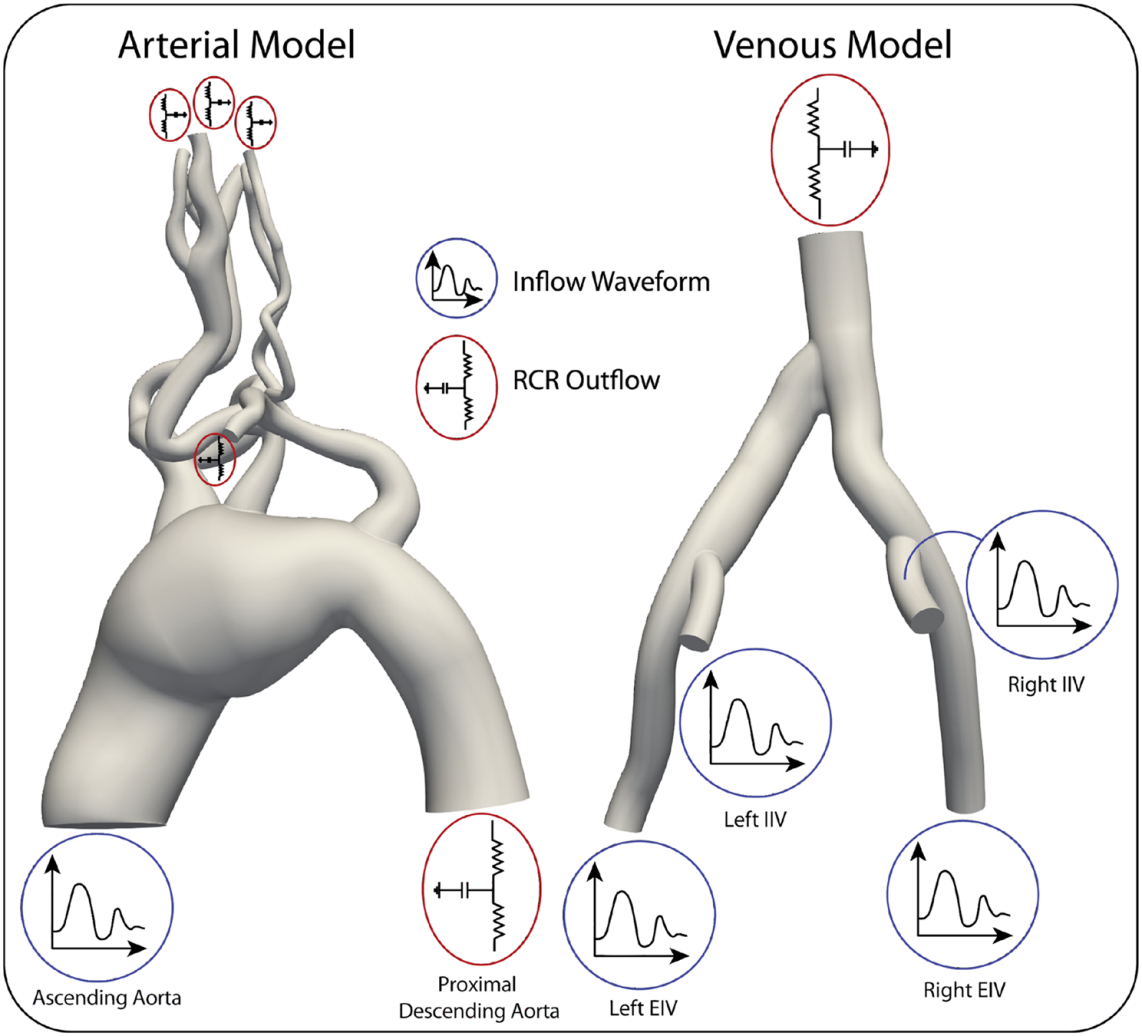
Computational domains of the patient-specific arterial model (Left) and venous model (Right). Inflow and outlet boundary conditions are specified as either a prescribed inflow waveform or reduced order Windkessel model.

A patient-specific thoracic aortic aneurysm model was generated from computed tomography angiography (CTA) image data using CRIMSON^33^. Figure 1 (left panel) shows the computational domain, consisting of the ascending and proximal descending aorta and 9 outlet branches, and a schematic of the boundary conditions. An echocardiography-derived periodic flow waveform (time period $T=0.91\,\mathrm {s}$) was mapped to a parabolic velocity profile, and prescribed as the inflow condition at the inlet face of the aortic model. This corresponds to a maximum Reynolds number of approximately $\mathrm {Re_{max}}=2.1 \times 10^3$ (based on Newtonian blood viscosity value). Three-element Windkessel models^34^ were prescribed at the outlet faces with parameters listed in Table S1 of Electronic Supplementary Information^8^. A zero velocity boundary condition was prescribed on all walls.

Several meshes with increasing levels of refinement were considered. A mesh with 22 million linear tetrahedral elements and 3.9 million nodes was ultimately chosen to adequately capture flow recirculation in the aneurysmal region. Cycle-to-cycle periodicity was achieved after four cardiac cycles, corresponding to a physical time of $t = 3.64~\mathrm {s}$.

#### Venous model

A patient-specific venous model was generated from CTA image data using CRIMSON^33^. Figure 1 (right panel) shows the computational domain, consisting of the: inferior vena cava (IVC), left common iliac vein (left CIV), left internal iliac vein (left IIV), left external iliac vein (left EIV), right common iliac vein (right CIV), right internal iliac vein (right IIV), and right external iliac (right EIV) vein , and a schematic of the boundary conditions. The venous geometry was scaled to match literature diameters values of the IVC and iliac veins.

Measurements of velocity were obtained at the IVC, common, and external iliac veins using duplex Doppler ultrasonography. Average flow values were calculated based on the mean velocity and diameter values obtained from the CTA. Internal iliac vein flows were deduced from the difference between the common and external iliac flows. A period of $T=0.8\,\mathrm {s}$ was utilized for all waveforms, which were mapped to a parabolic velocity profiles prescribed at the 4 inlet vessel faces of the model (e.g., external and internal iliac veins), resulting in a maximum Reynolds number of approximately Re$_{\mathrm {max}} = 405$ (based on Newtonian blood viscosity value). A three-element Windkessel model was applied to the IVC outlet face with parameters listed in Table S2. A zero velocity boundary condition was prescribed on all walls.

Several meshes with increasing levels of refinement were considered. Reported results correspond to a mesh consisting of 2 million nodes and 12.5 million linear tetrahedral elements. Cycle-to-cycle periodicity was achieved after four cardiac cycles, corresponding to a physical time of $t = 3.2~\mathrm {s}$.

## Results

### Verification of power-law implementation

To verify our implementation of the shear-dependent non-Newtonian viscosity models, we compared the numerical results for the Power-Law model against an analytical solution in an idealized cylindrical geometry. To this end, we considered a cylindrical domain with diameter $d = 40.0~\text {mm}$ and length $l = 200.0~\mathrm {mm}$. Using a Power-Law constitutive model, a steady flow solution was obtained by prescribing a constant flow rate of $833.33~\text {mm}^3/\mathrm {s}$ mapped to a Poiseuillle parabolic velocity profile at the inlet, with a maximum centerline velocity $\text {v}_\text {max}=1.3~\text {mm}/\text {s}$ and a maximum Reynolds number $\text {Re}_\text {mean}=8.0$. No-slip and zero traction boundary conditions were prescribed on the lateral wall and the outlet face, respectively.

Our numerical implementation was observed to be in excellent agreement with the analytical Poiseulle profile, as shown in Fig. S2 of the Electronic Supplementary Information.

### Patient-specific hemodynamic analysis

In this section, diastolic solutions for velocity, vorticity, and WSS are discussed for both the Newtonian and Carreau-Yasuda constitutive laws, for the arterial and venous models. Vorticity is defined as the curl of the velocity field ($\mathbf {\omega } = \mathbf {\nabla } \times {{\textbf {u}}}$) and describes the local spinning motion of the fluid. To provide a quantitative comparison between solutions, surface (in-plane velocity and WSS) or volume (vorticity) averages of the solution fields are calculated at four locations: mid-aneurysm (section A) and distal aorta (section B) for the aortic model, and IVC (section C) and left common iliac vein (section D) for the venous model. Furthermore, to study the differences between solutions on a point by point basis, we define a relative difference metric as:

9 $$\begin{aligned} \text {Relative Difference} = \frac{\left\Vert \text {Newtonian{-}Carreau Yasuda}\right\Vert }{\overline{\left\Vert \text {Carreau Yasuda}\right\Vert }}, \end{aligned}$$

where $\left\Vert \cdot \right\Vert$ denotes the L2 norm of an Eulerian field, and ${\overline{\left\Vert \text {Carreau Yasuda}\right\Vert }}$ is a reference mean value of the Carreau-Yasuda solution, calculated for each slice of the in-place velocity, and for the shaded regions in the ascending aorta and IVC for the vorticity and WSS (see Figs. 3 and 4). This reference mean value defines a suitable norm to study relative differences for each solution field between Newtonian and Carreau-Yasuda models^2^.

#### In-plane velocity

**Figure 2:**
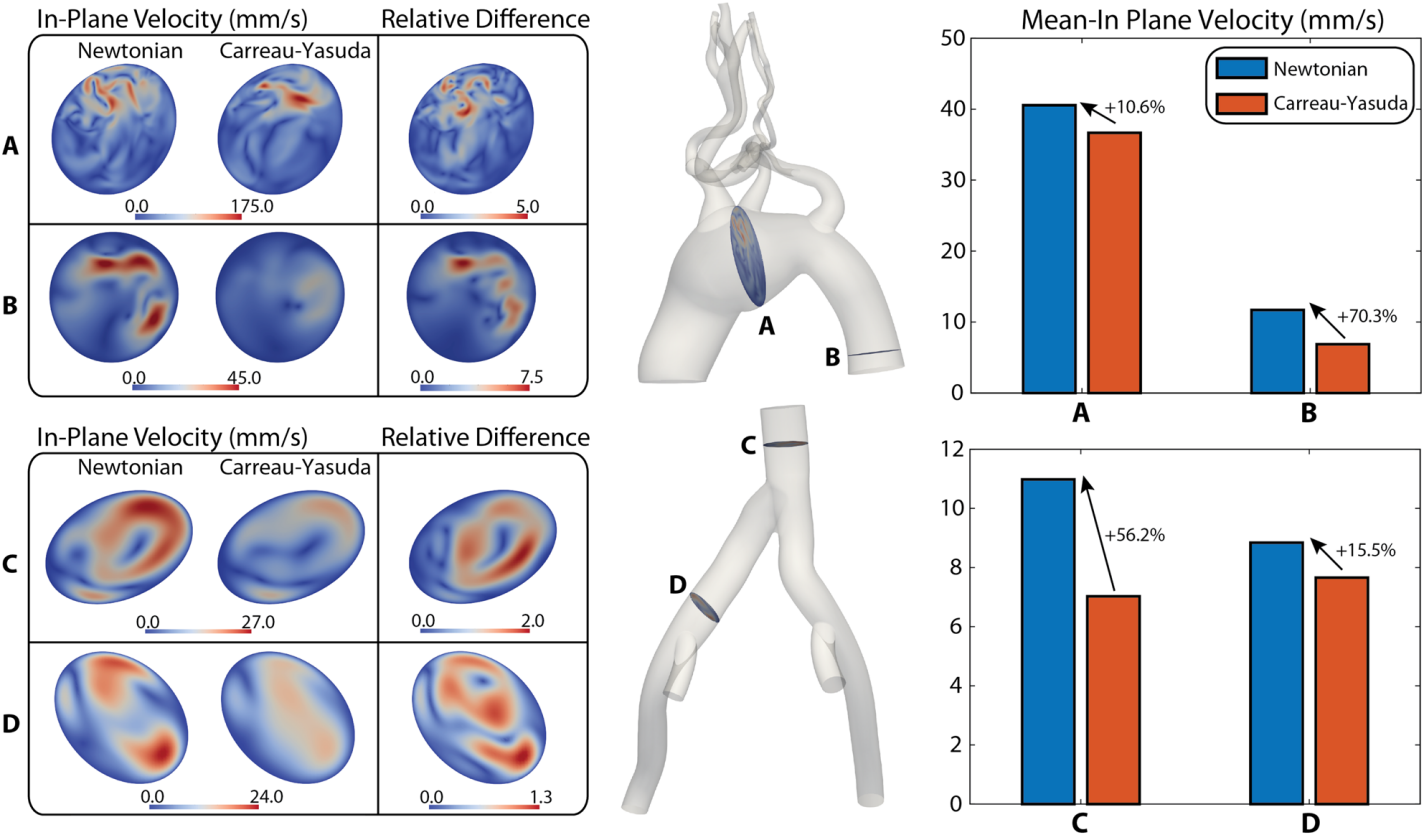
Arterial and venous in-plane velocity in diastole. (Left) Contour plots of in-plane velocity magnitude for the Newtonian and Carreau-Yasuda models, and relative difference between the two, at four representative locations. (Right) Bar plots of mean values for each location.

Figure 2 shows contour plots of the in-plane velocity magnitude and bar plots of mean in-plane velocity. In the arterial model, larger mean values of in-plane velocities are observed in the Newtonian model, with larger discrepancies between models seen in section B (Section A: $10.6\%$ and Section B: $70.3\%$). Larger relative differences in in-plane velocity magnitude are also observed in section B with relative differences of up to 7.5. In the venous model, similar patterns are observed: larger mean in-plane velocities are obtained with the Newtonian model (Section C: $56.2\%$ and Section D: $15.5\%$), and larger relative differences in in-plane velocity magnitude are seen in Section C. (Max. Relative Difference 2). Due to the smaller pulsatility and lower flow in the venous model, smaller values of in-plane velocity are obtained (max. in-plane velocity 27 mm/s) relative to the arterial model (max. in-plane velocity 175 mm/s).

#### Vorticity

**Figure 3:**
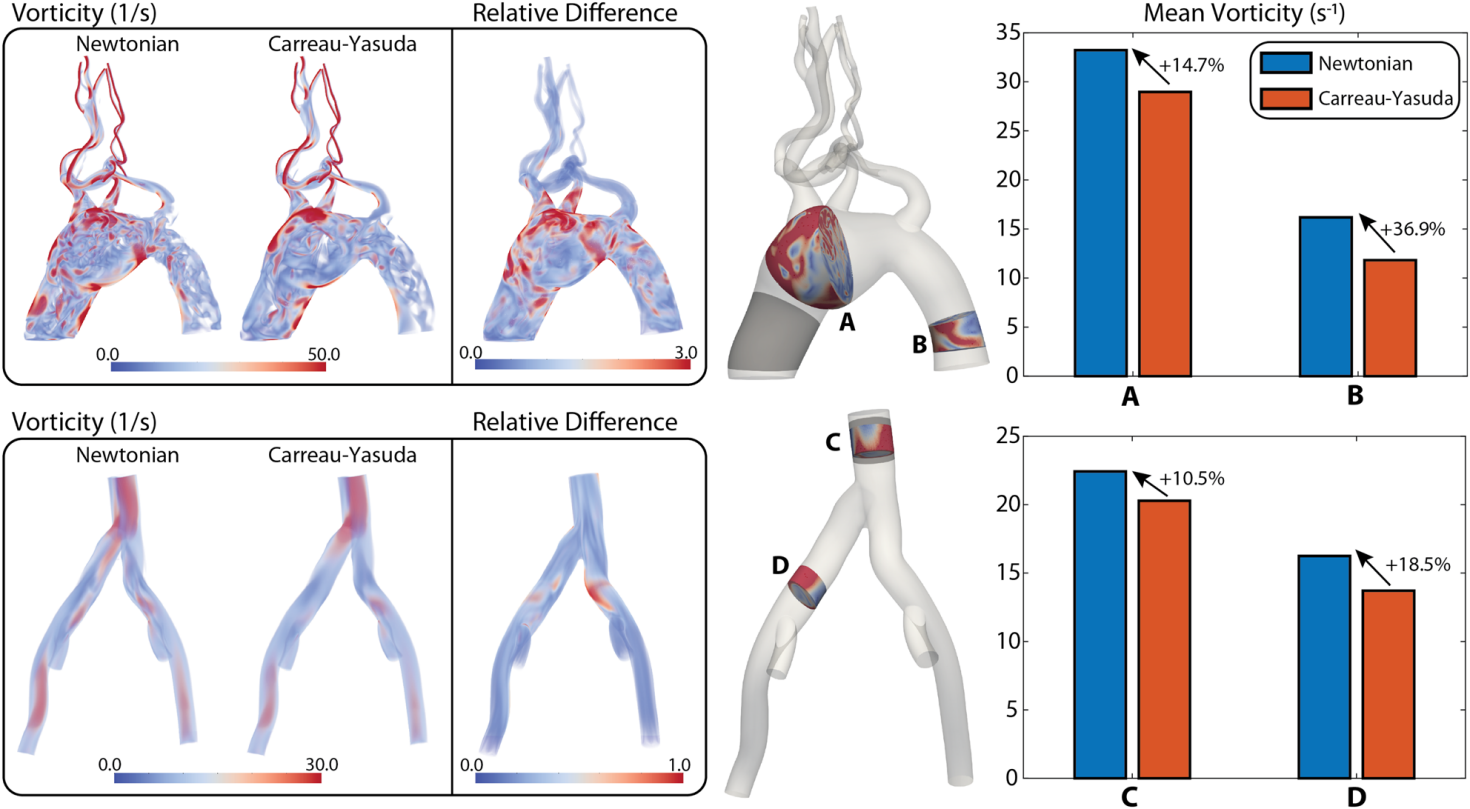
Arterial and venous vorticity in diastole. (Left) Volume rendering plots of vorticity magnitude for the Newtonian and Carreau-Yasuda models, and relative difference between the two. (Right) Bar plots of mean values for four representative locations (**A**–**D**).

Figure 3 shows volume rendering plots of vorticity magnitude and bar plots of mean vorticity. More vortical structures are apparent in the Newtonian solution for both the arterial and venous models, with a maximum relative difference of 3.0 in the arterial and 1.0 in the venous model. Mean vorticity is larger in all four locations for the Newtonian model, ranging from 10.5% in the IVC (Section C) to 36.9% in the descending aorta (Section B). Due to the lower pulsatility of the venous flow, smaller values of vorticity are obtained in the venous model (max. vorticity 30 $\text {s}^{-1}$) relative to the arterial model (max. vorticity 50 $\text {s}^{-1}$).

#### Wall shear stress

**Figure 4:**
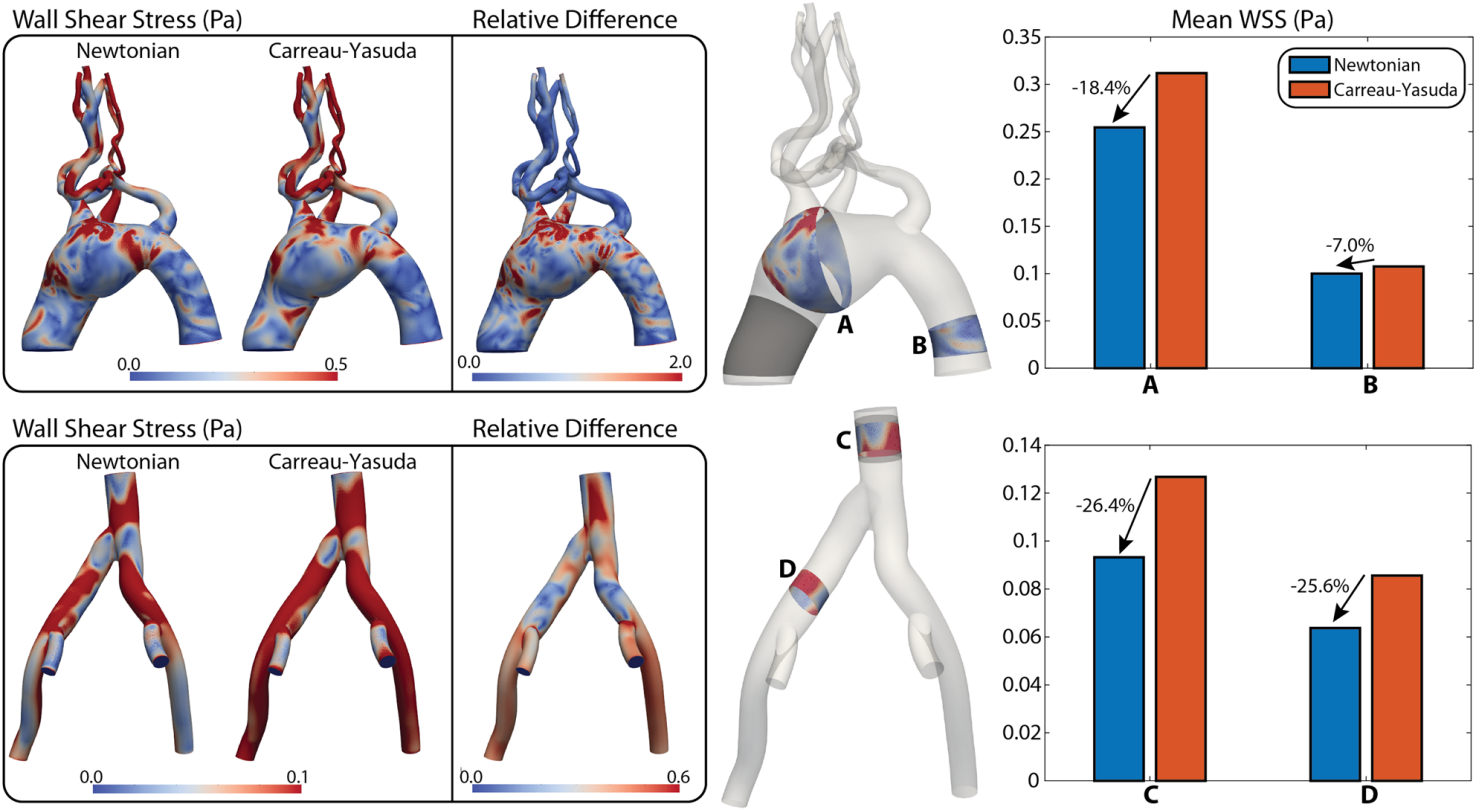
Arterial and venous WSS in diastole. (Left) Contour plots of WSS magnitude for the Newtonian and Carreau-Yasuda models, and relative difference between the two. (Right) Bar plots of mean values for four representative locations (**A**–**D**).

Figure 4 shows contour plots of WSS magnitude and bar plots of mean WSS. In the arterial model, mean WSS is smaller in the Newtonian solution (18.4% smaller in the mid-aneurysm and 7.0% smaller in the descending aorta) in both the aneurysm and descending thoracic aorta. For the venous model, smaller values of WSS are also obtained with the Newtonian solution (26.4% smaller in the IVC and 25.6% smaller in the left common iliac vein). Larger relative differences are observed in the arterial model (2.0 for the arterial model vs. 0.6 for the venous model). Due to the smaller pulsatility of the venous flow, smaller values of WSS are obtained in the venous model (max. WSS 0.10 Pa) relative to the arterial model (max. WSS 0.50 Pa). The observed venous WSS values are in line with the infrarenal IVC venous WSS values reported in Cheng et al. $0.2 \pm 0.06$ Pa for healthy individuals^35^, although slightly below their reported range.

We remark that the reported trends in in-plane velocity, vorticity, and WSS are consistent throughout the cardiac cycle for each model, and not just in diastole.

#### Regions of critical shear rate

**Figure 5:**
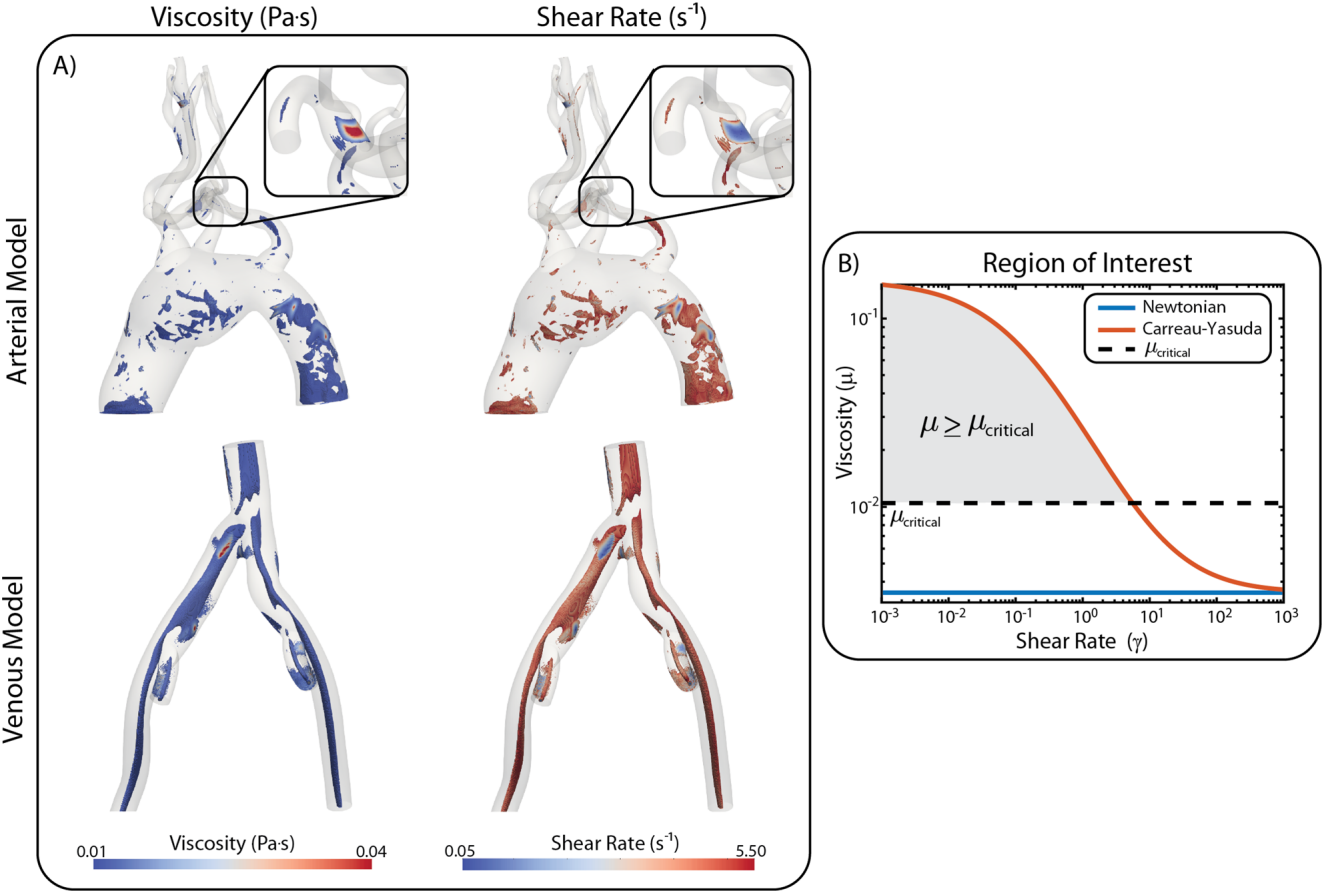
(**A**) Maps of viscosity and shear rate obtained with the Carreau-Yasuda model in the regions of the computational domain where the viscosity is greater than the critical threshold ($\mu \ge \mu _\text {critical})$. These are regions where the non-Newtonian effects are most important. (**B**) Critical viscosity is defined by $\mu _\text {critical} = 3\mu _\text {N}$.

Lastly, regions of high viscosity and low shear rate in the Carreau-Yasuda model are identified through an arbitrary threshold of viscosity: ($\mu \ge \mu _\text {critical} = 0.01\,\text {Pa.s}=3\mu _\text {N}$), see Fig. 5, for both the arterial and venous models in diastole. These are the regions where the non-Newtonian effects can be considered to have the most impact on the solution fields.

A few areas of increased viscosity can be identified in the aneurysmal and descending aortic regions, as well as in the head and neck vessels of the aortic model. In contrast, the venous model shows extensive regions of elevated viscosity and low shear rate throughout the entire model, except in the near wall regions. In the arterial model, regions of low shear rate are expected in the aneurysmal region, but it is interesting to also observe those regions in the descending aorta, where the aortic diameter is normal. In the venous model, widespread areas of low shear rate emphasize the need to consider non-Newtonian viscosity models when studying problems on the large veins.

### Patient-specific transport analysis

The transport of biochemical species such as proteins, platelets and chemical signaling species plays a significant role in the initiation and propagation of various cardiovascular diseases such as thrombosis and atherosclerosis^25,36,37^. We studied the impact that the choice of constitutive law has on two different models of transport: Lagrangian particle tracking, and scalar advection-diffusion (AD) equations in both anatomical models.

#### Lagrangian particle tracking

Approximately one million massless particles were injected into the arterial and venous anatomical models and tracked for ten cardiac cycles. For the arterial model, a single bolus was released at the ascending thoracic aorta. In the venous model, four boluses of approximately 250,000 particles each were released at the left EIV, left IIV, right EIV, and right IIV. Particles were tracked as they were passively advected through each computational domain over time and collected at the outflow faces. Statistics on PLAP and the number of particles were recorded.

**Figure 6:**
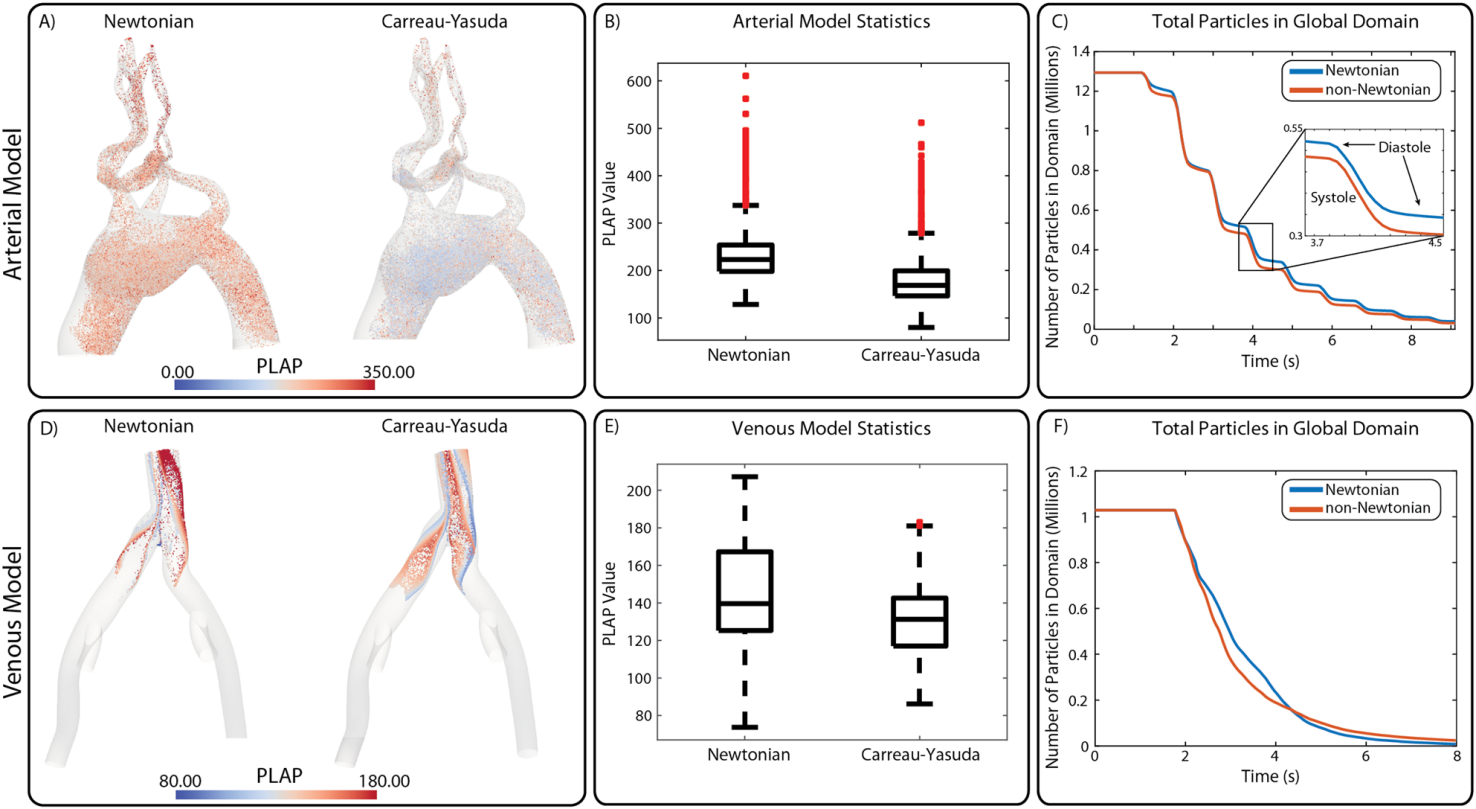
(**A**,**D**) Particles left in the arterial and venous computational domains for Newtonian and Carreau-Yasuda simulations. (**B**,**E**) Box and whisker plot describing the particles left in the domains. (**C**,**F**) Line plots describing the number of particles in the computational domains over time.

Figure 6A,D shows the particles remaining in the arterial and venous models after ten cardiac cycles. Mean PLAP values in both models are larger in the Newtonian analysis compared to the Carreau-Yasuda, see Fig. 6B,E.

Figure 6C,F show the total number of particles inside the anatomical domains over time. Particles leave the domains at different rates due to differences in viscosity. Interestingly, particles leave the domains at a slower rate in the Newtonian model. This is likely due to the increased vorticity of the flow in the Newtonian simulation, see Fig. 3. The pulsatility of the arterial flow is apparent in panel C, where we observe that non-Newtonian viscosity is more important during diastole (flat portions of the curve). After $t = 3.64$ s, the differences between Newtonian and Carreau-Yasuda simulations are apparent throughout the entire cardiac cycle. For the venous model (panel F), particles leave the domain at a relatively constant rate due to the smaller pulsatility of the flow.

#### Scalar mass transport via advection-diffusion (AD)

Next, we perform a scalar AD mass transport analysis using our previously implemented numerical framework within CRIMSON^32^. For all mass transport simulations, we investigate the spatio-temporal evolution of species injected through the inlet face(s) of the computational domains. The goal is to understand whether the choice of blood constitutive model renders differences in the lumen and near-wall scalar concentration field(s), since it is well known that initiation and propagation of numerous cardiovascular diseases (e.g., thrombosis, atherosclerosis, etc.) are influenced by near-wall transport^36–38^. An initial concentration of $c=0 ~\text {mol}/\text {mm}^3$ is assumed for all species and a constant concentration of $c=10 ~\text {mol}/\text {mm}^3$ is prescribed at the inlet face(s) for each species. A zero total flux boundary condition is prescribed at all walls while a consistent flux boundary condition is prescribed at the outlet face(s)^32^. A constant diffusion coefficient $D = 1.0\,\mathrm {mm}^2/\mathrm {s}$ is used for all species.

**Figure 7:**
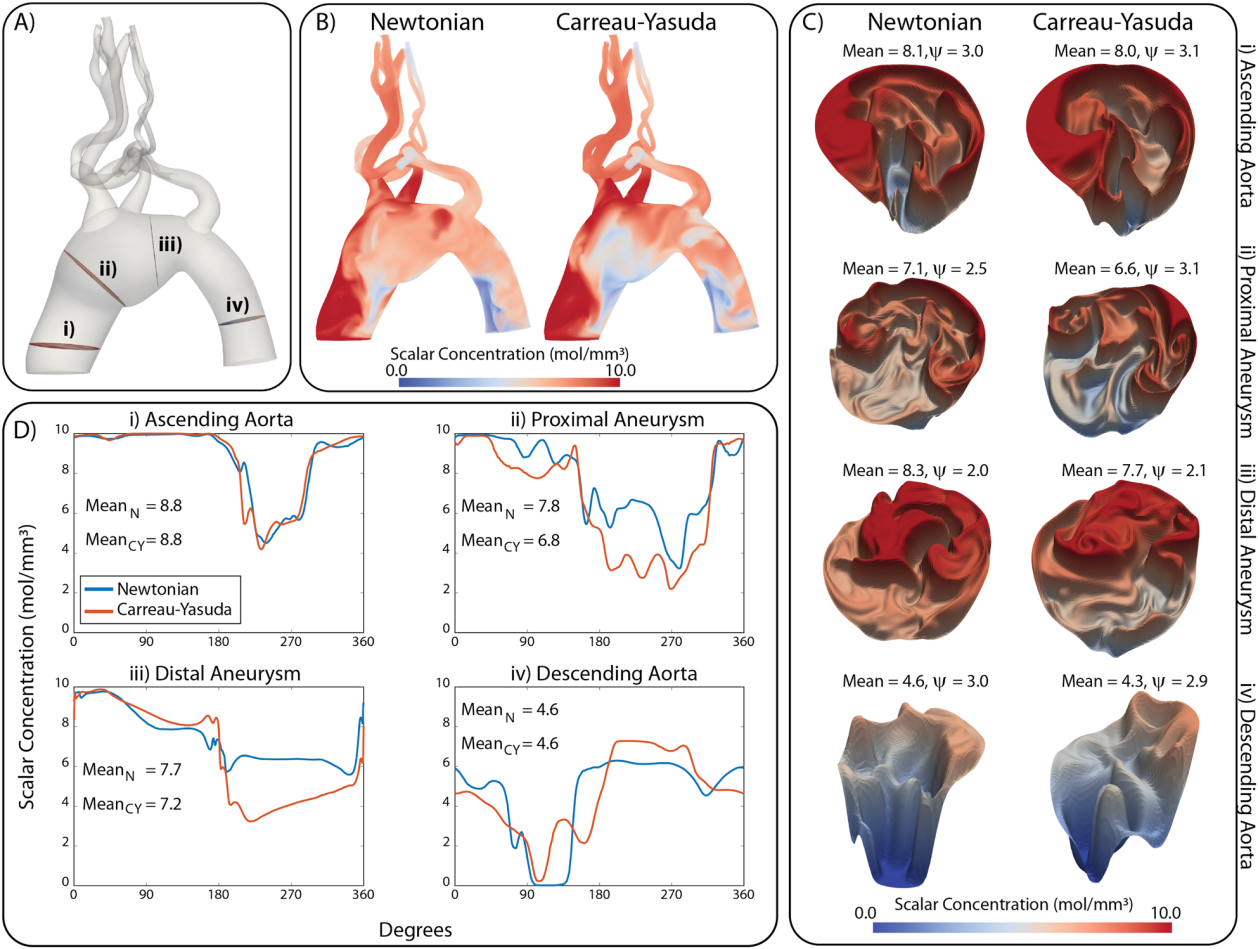
(**A**) Arterial computational domain highlighting four locations (i–iv) along the aortic arch. (**B**) Volume rendering of the scalar field for the Newtonian and non-Newtonian solutions after three cardiac cycles (time $t=2.92\mathrm {s}$). (**C**) Warp visualization of the scalar field at locations (i–iv). (**D**) Scalar concentration along the aortic wall at locations (i–iv) for Newtonian and non-Newtonian solutions.

Figure 7A shows the arterial computational domain with four locations (i)–(iv) highlighted along the aorta. Figure 7B shows a qualitative comparison between volume rendered plots of scalar concentration for the Newtonian and Carreau-Yasuda solutions during diastole. Larger discrepancies between scalar fields are apparent within the aneurysmal region and descending aorta. Figure 7C shows warp plots of luminal scalar concentration at locations (i)–(iv). For each location, We report the average value of the scalar concentration, as well as a *tortuosity index* $\psi$, defined as the ratio of the surface area of the warp plot over the luminal surface area. The Newtonian model produced larger mean values of scalar concentration for all four locations, with the largest differences observed in locations (ii) and (iii), the farthest away from the boundaries. Furthermore, the tortuosity index was larger for the Carreau-Yasuda model in all locations except the near outlet location (iv). This points to a larger heterogeneity in the scalar field, with many more ups and downs in the solution, despite the overall lower mean values of scalar compared to the Newtonian case.

Lastly, Fig. 7D shows near-wall scalar concentration for the Newtonian and Carreau-Yasuda solutions at locations (i)–(iv). The scalar concentration is plotted against an angular position describing the location along the aortic wall. Significant differences are observed in the concentration profiles at location ii (7.8 vs. 6.8 mol/mm^3^, 14.7%). Equivalent mean concentration values were obtained at the ascending and descending aorta (difference < 1%) and minor differences (6.3%) were observed at the distal aneurysm. In both the proximal and distal aneurysm the mean scalar concentrations are lower in the Carreau-Yasuda solution along the majority of the vessel wall, resulting in lower mean values compared to the Newtonian solution.

**Figure 8:**
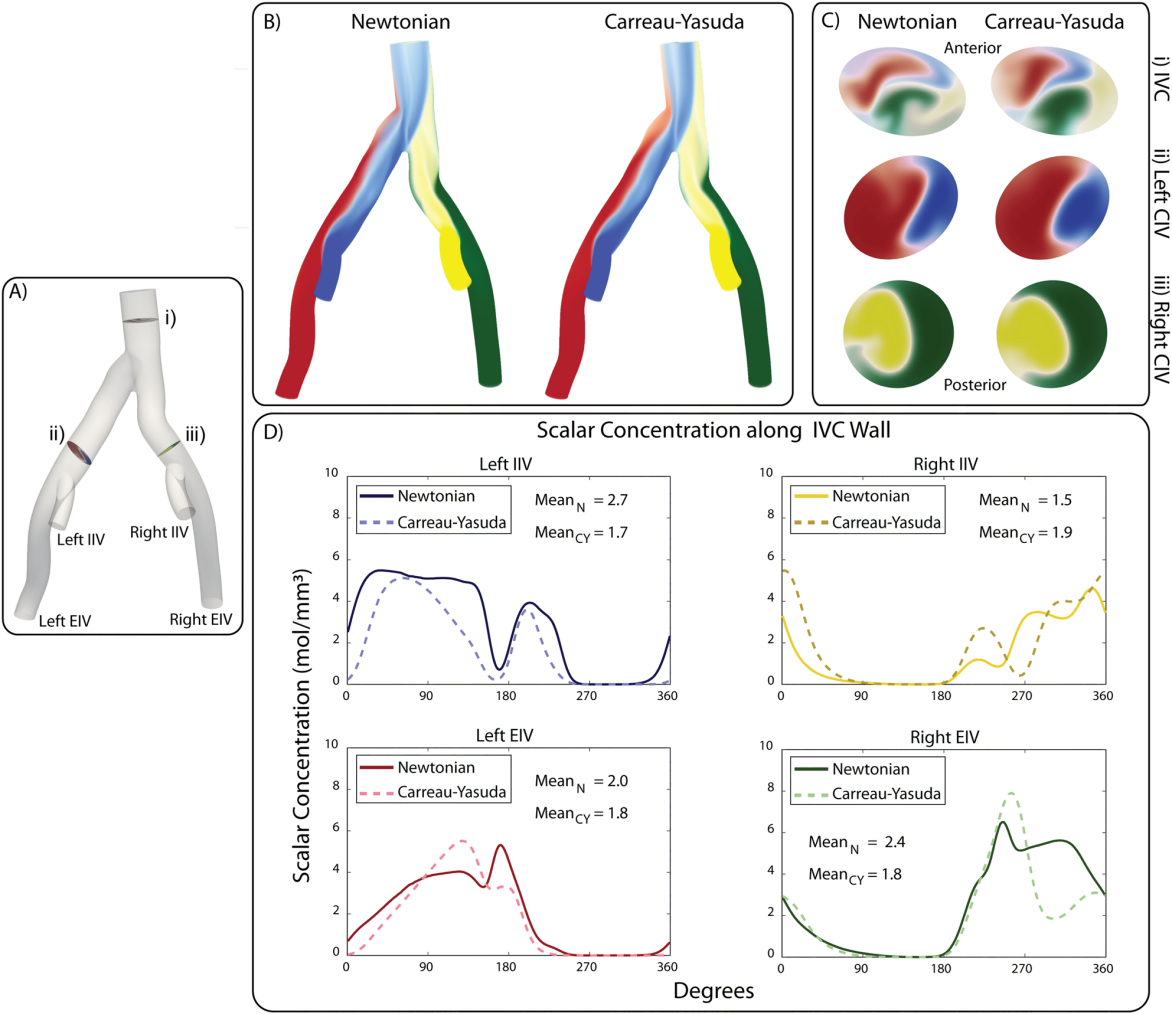
(**A**) Venous computational domain highlighting three locations (i–iii). (**B**) Volume rendering of the four scalar scalar fields for the Newtonian and non-Newtonian simulations. (**C**) Concentration contours of the four scalar fields at locations (i–iii). (**D**) Scalar concentration along the IVC wall for Newtonian and non-Newtonian simulations for each scalar species. All results after eight cardiac cycles (time $t=6.4\;\mathrm {s}$).

Figure 8A shows the venous anatomical model with three highlighted locations at which a detailed analysis of the different concentration fields $c_{j}$ is performed: (i) IVC, (ii) left CIV, and (iii) right CIV. Figure 8B shows volume rendered plots of scalar concentrations for the Newtonian and Carreau-Yasuda solutions in diastole. Figure 8C shows contour plots of scalar concentration for each species at the three locations highlighted in Panel A. The plot at the IVC (i) shows mixing of the four scalars with significant qualitative differences between the Newtonian and Carreau-Yasuda solutions. The Carreau-Yasuda solution is characterized by smoother, more diffused concentration profiles. This finding is consistent with the higher viscosity in the Carreau-Yasuda solution at lower shear rates which diffuses gradients in the flow field. The concentration plots at the left and right CIV (ii, iii) also show subtle qualitative differences. For both solutions, it is interesting to observe that the anterior aspect of the IVC wall is predominantly perfused by scalars transported from the left side (left EIV and left IIV) and the posterior aspect by scalars originating on the right side (right EIV and right IIV).

Lastly, Fig. 8D compares the concentration of the Newtonian and Carreau-Yasuda solutions along the IVC wall for each scalar species injected at the left EIV, right EIV, left IIV, and the right IIV . The scalar concentration is plotted against an angular distance describing the location along the IVC wall. Significant differences in wall concentration for each scalar are observed, ranging from 10.7% for the Left EIV , to 64.0% for the Left IIV . Interestingly, the mean value of the Carreau-Yasuda solution is lower than that of the Newtonian solution for every location except the Right IIV. This is likely due to the decreased vorticity (seen in Fig. 3) and the decreased residence time (see Fig. 6) of the Carreau-Yasuda solution.

## Discussion

Despite the long-standing knowledge of the shear thinning behavior of blood, computational studies have typically assumed a shear-independent Newtonian viscosity model for blood. While this assumption is generally applicable in large arteries characterized by high shear rates, it is difficult to justify in regions of vasculature exhibiting low shear rates such as veins and diseased arteries. Moreover, previous studies which considered non-Newtonian viscosity often employed idealized vascular geometries^11^, despite the evident role that complex anatomical features have in altered flow patterns and have not investigated the effects of non-Newtonian viscosity on transport phenomena.

The current study combines our recent advances in the modeling of cardiovascular hemodynamics^32^ and contains several novel elements such as the use of patient-specific venous geometries, efficient stabilization strategies, and investigation of both Lagrangian and Eulerian metrics of flow transport. We remark that this study is a part of our larger ongoing effort to develop robust and efficient computational tools for cardiovascular hemodynamics and transport. In this paper, we set out to investigate the impact that the choice of constitutive model has on hemodynamic metrics (velocity, vorticity, wall shear stress) as well as transport metrics (scalar and Lagrangian based) for arterial and venous anatomies in which low shear and recirculation are expected.

We first implemented two shear-dependent non-Newtonian viscosity models of blood and demonstrated excellent agreement between profiles (Fig. S2). We note that our numerical implementation of non-Newtonian viscosity models was explicit (i.e., we only accounted for shear-dependent viscosity on the right-hand-side residual and not the left-hand-side linearization matrix). This explicit implementation of shear-dependent viscosity yielded good convergence behavior in the numerical analyses and led to minor increments in computational cost compared to solutions obtained with a Newtonian model. Given this implementation approach, incorporation of additional constitutive models will be straightforward, requiring minimal coding effort.

We then studied the effect of blood rheology in two patient-specific models which are expected to exhibit regions of low shear rate: a thoracic aortic aneurysm model, and a venous model of the IVC and iliac veins, see Fig. 1. In each case, we performed a comparative hemodynamics and transport analysis using Newtonian and Carreau-Yasuda viscosity models.

For the arterial model, our analysis of hemodynamic indices revealed lower in-plane velocities and vorticity and larger WSS in the Carreau-Yasuda solution, see Figs. 2, 3, and 4. Lower vorticity in the Carreau-Yasuda solution is expected since non-Newtonian models are characterized by higher viscosity and lower shear rates that result in larger diffusion on the flow vorticity. In the Carreau-Yasuda solution, the viscosity is sufficiently large to result in greater WSS.

The venous model was characterized by lower pulsatility and velocities compared to the arterial model. Our analysis again revealed lower velocities and vorticity (Figs. 2 and 3) and larger WSS (Fig. 4) in the non-Newtonian case. This observation suggests that the increased viscosity in the non-Newtonian case is sufficiently larger to result in higher WSS, despite the lower velocity gradients.

A Lagrangian particle tracking analysis was then performed in both the arterial and venous models to assess the effect of blood rheology on cardiovascular mass transport (Fig. 6). Our analysis revealed an increase in mean PLAP in the Newtonian case for both arterial and venous models (Fig. 6B,E). In addition, non-Newtonian viscosity affected the rate at which particles left the computational domain with particles leaving at a slower rate in the Newtonian case (Fig. 6C,F). We hypothesize that this is caused by the increased vorticity (see bar plots in Fig. 3) which led to particles recirculating longer before exiting the domain. Residence time and PLAP are two metrics that have both been linked to thrombosis formation^29,39^ and this suggests that considering accurate blood rheological models can have a significant effect on solutions for computational cardiovascular disease models.

Lastly, in Figs. 7 and 8 our mass transport analysis revealed significant differences in the concentration fields between the Newtonian and Carreau-Yasuda solutions for both the arterial and the venous models. In the arterial model, the greatest differences in both bulk and near-wall transport were observed in the proximal aneurysm (location ii) where the mean near-wall concentration value differed by 14.7% and the surface area of the bulk concentration profile differed by 20.3%. In the venous model, the greatest differences in bulk and near-wall transport were observed in the IVC where the four scalars mix. Significant differences in wall concentration values were observed ranging from 10.7% for $c_{j} = 2$ (Left EIV), to 64.0% for $c_{j} = 4$ (Left IIV). Interestingly, the mean value of the Carreau-Yasuda solution was lower than the Newtonian solution for every location except the Right IIV. This is presumably due to the decreased vorticity and residence time of the Carreau-Yasuda solution, see Figs. 3 and 6 which increases the rate at which the scalar concentration exits the domain. The differences observed both in the bulk domain and perhaps more importantly, in the near-wall region, again highlight the importance of considering non-Newtonian rheological models to obtain accurate assessment of mass transport phenomena.

The AD analysis was performed over a finite number of cardiac cycles under periodic flow conditions: 4 for the arterial case and 8 for the venous case. This means the solutions for the scalar field were still developing and had not reached a cycle-to-cycle periodic state. The reported transient scalar analysis could be relevant to study processes such as drug delivery and thrombosis initiation, where a finite concentration is delivered into the system over a relatively short period of time. Future work should address studying the effects of non-Newtonian viscosity on cycle-to-cycle periodic scalar mass transport solutions.

One of the primary limitations of the current study is that both the arterial and venous walls were modeled as rigid. The venous walls, in particular, are known to exhibit significant compliance, rendering this assumption as questionable. However, owing to the limited availability of vessel wall material properties and their regional variation, the incorporation of vessel wall compliance in patient-specific venous anatomies demands a separate focused effort and will be the subject of future work.

The present study is also limited to two patient-specific geometries: one arterial and one venous. Future studies should aim at performing similar analyses in additional patient-specific geometries to draw further conclusions if the trends observed are consistent among a larger patient population.

In this study, we used previously reported values of viscosities in the low and high shear rate limit for the Carreau-Yasuda model^3,7,25,26^. However, there exist numerous other computational studies^40–42^ that employed different values, making it difficult to compare results across studies. Therefore, better standardization and computational benchmarks are needed, perhaps in idealized geometries, to allow both the validation of new computational frameworks and the study of isolated effects of flow conditions and vessel geometry. It is also important to note that the Power-Law model begins to fail at both very high and low shear rates, where the estimated value trends toward infinity rather than reaching a constant value as observed experimentally. This limits the usefulness of the Power-Law model compared to other constitutive models such as the Carreau-Yasuda.

Future studies should investigate shear-dependent rheological models in healthy arterial and/or diseased venous models which may have considerably fewer regions of low shear flow, as well as study other well known constitutive models for blood such as the Casson and Herschel-Bulkley.

## Supplementary Information

Supplementary Information.

## Data Availability

All data generated or analyzed during this study are included in this published article (and its Supplementary Information files).
