# Supplementary material for: Effects of non-Newtonian viscosity on arterial and venous flow and transport
Source: Sci Rep. 2022 Nov 29;12:20568. doi: 10.1038/s41598-022-19867-1 (PMC9709089; doi:10.1038/s41598-022-19867-1)
Supplement: Supplementary file 1 — Supplementary Information. [file 41598_2022_19867_MOESM1_ESM.pdf]

Supplemental Material

Effects of non-Newtonian Viscosity on Arterial and Venous Flow and Transport

Sabrina R. Lynch, Nitesh Nama, C. Alberto Figueroa

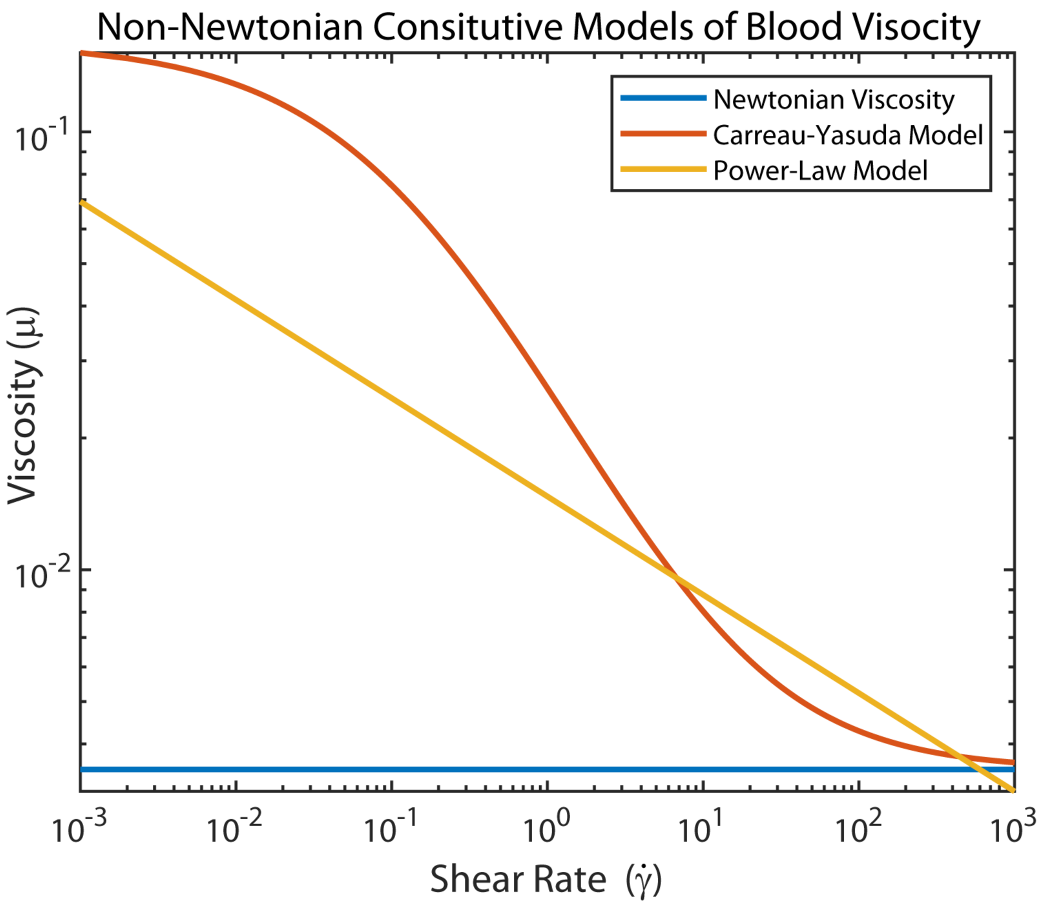

Figure 1: Constitutive Models

1 Verification against analytical solution

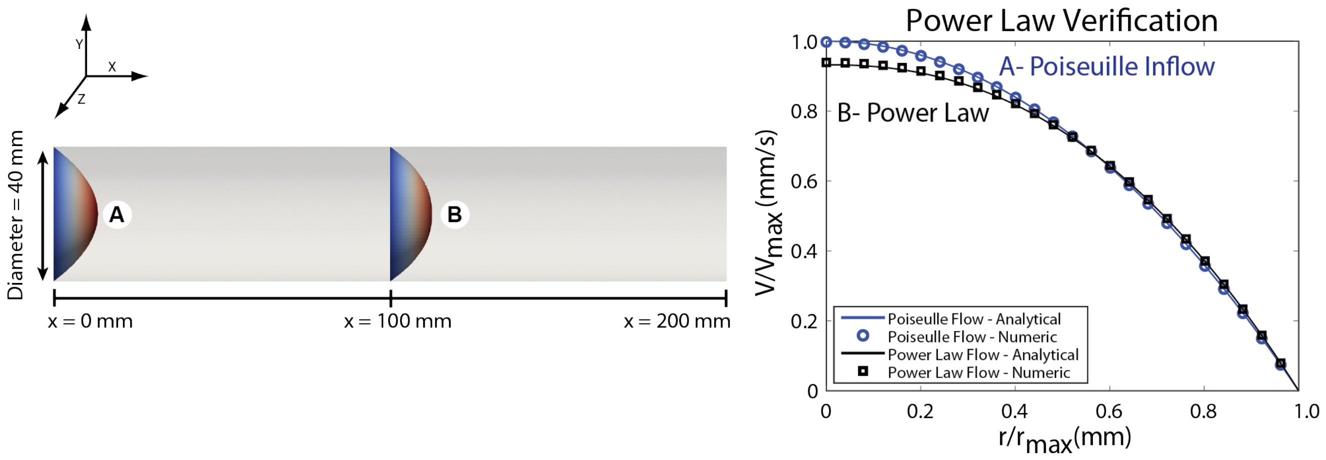

Figure S2: Verification of a Power-Law model. Left: Cylinder dimensions. The prescribed Poiseuille profile at the inlet (A) develops into a non-Newtonian Power-Law velocity profile at the mid-section of the cylinder (B). 1D plots show good agreement between analytical (black solid line) and numerical (black symbols) solutions at section B (max. error: 0.72%).

Figure S2 (left) shows 3D warps of the prescribed velocity profile at the inlet (section A) and the numerically obtained velocity profile at the midplane of the domain (section B). Figure S2 (right) compares the analytical and numerical solution at these two locations. The prescribed Poiseuille profile at the inlet (blue circles) develops into a non-Newtonian profile along the length of the cylinder, and by its mid-section (black solid symbols) shows an excellent agreement (max. error: 0.72%) with the Power-Law analytical solution (black solid line).

2 Three-element Windkessel parameter values

2.1 Aortic Model

**Branch**	**Proximal Resistance ($g/(mm^4 \cdot s)$)**	**Capacitance ($mm^4 \cdot s^2/g$)**	**Distal Resistance ($g/(mm^4 \cdot s)$)**
RSA	0.19	0.15	2.29
REC	0.63	0.11	7.09
LEC	0.28	0.10	3.28
TA	0.01	5.18	0.22
LSA	0.18	0.15	2.27
LIC	0.19	0.18	2.36
RIC	0.17	0.23	2.19
RVA	0.54	0.09	6.03
LVA	0.44	0.07	4.80

Table S1: Three-element Windkessel values used for aortic model.

RSA- Right subclavian artery
REC - Right external carotid artery
LEC - Left external carotid artery
TA -Thoracic Aorta
LSA- Left subclavian artery
LIC- Left internal carotid artery
RIC- Right internal carotid artery
RVA- Right vertebral artery
LVA- Left vertebral artery

2.2 Venous Model

**Outlet**	**Proximal Resistance ($g/(mm^4 \cdot s)$)**	**Capacitance ($mm^4 \cdot s^2/g$)**	**Distal Resistance ($g/(mm^4 \cdot s)$)**
IVC	0.0033	3.4	0.063

Table S2: Three-element Windkessel values used for venous model of the inferior vena cava and iliac veins.

IVC- Inferior vena cava
